# Automated Assessment of Peristomal Skin Discoloration and Leakage Area Using Artificial Intelligence

**DOI:** 10.3389/frai.2020.00072

**Published:** 2020-09-10

**Authors:** Niels K. Andersen, Pernille Trøjgaard, Nana O. Herschend, Zenia M. Størling

**Affiliations:** ^1^VENZO.Nxt, Copenhagen, Denmark; ^2^Coloplast A/S Denmark, Humlebaek, Denmark

**Keywords:** artificial intelligence, peristomal skin complications, leakage, discoloration, ostomy, convolutional neural networks

## Abstract

For people living with an ostomy, development of peristomal skin complications (PSCs) is the most common post-operative challenge. A visual sign of PSCs is discoloration (redness) of the peristomal skin often resulting from leakage of ostomy output under the baseplate. If left unattended, a mild skin condition may progress into a severe disorder; consequently, it is important to monitor discoloration and leakage patterns closely. The Ostomy Skin Tool is current state-of-the-art for evaluation of peristomal skin, but it relies on patients visiting their healthcare professional regularly. To enable close monitoring of peristomal skin over time, an automated strategy not relying on scheduled consultations is required. Several medical fields have implemented automated image analysis based on artificial intelligence, and these deep learning algorithms have become increasingly recognized as a valuable tool in healthcare. Therefore, the main objective of this study was to develop deep learning algorithms which could provide automated, consistent, and objective assessments of changes in peristomal skin discoloration and leakage patterns. A total of 614 peristomal skin images were used for development of the discoloration model, which predicted the area of the discolored peristomal skin with an accuracy of 95% alongside precision and recall scores of 79.6 and 75.0%, respectively. The algorithm predicting leakage patterns was developed based on 954 product images, and leakage area was determined with 98.8% accuracy, 75.0% precision, and 71.5% recall. Combined, these data for the first time demonstrate implementation of artificial intelligence for automated assessment of changes in peristomal skin discoloration and leakage patterns.

## Introduction

Development of peristomal skin complications (PSCs) is the most common post-operative complication following creation of an ostomy (Meisner et al., 2012). PSCs are a constant challenge for a great majority of people living with an ostomy and various studies have reported that they affect 18–80% of the ostomy population (Burch, 2011; Fellows et al., 2017). The wide variation in incidence reporting of these complications may be due to the less systematic assessment of the peristomal skin together with the lack of availability to follow progression over time and between visits at the health care professionals. Hence, one of the challenges is to define the exact prevalence of PSCs and the underlying cause of the complication.

After the ostomy surgery, many people will experience changes in their peristomal area, and the height as well as the diameter of the ostomy may also change. This may require adjustment to their ostomy appliance to minimize the risk of having the peristomal skin exposed to output from the ostomy. Irritant contact dermatitis (ICD) is one of the main causes of PSCs (Herlufsen et al., 2006) and is induced by fecal output underneath the adhesive baseplate of the ostomy appliance also referred to as leakage. A visual clinical sign of ICD is redness of the peristomal skin. Suffering from leakage underneath the ostomy baseplate adds to the probability of the ICD diagnosis. A mild peristomal skin complication may progress into a more severe disorder if left unattended, and for this reason it can be beneficial to monitor the peristomal skin condition closely.

The Ostomy Skin Tool is current state-of-the art for assessing peristomal skin conditions. This tool allows nurses to have a standardized approach with a common language for making uniform evaluations of peristomal skin conditions at consultations (Martins et al., 2010). Despite the definite usefulness of the Ostomy Skin Tool, closely monitoring progression of PSCs is quite cumbersome as it requires regular dialogues and/or face to face meetings between the patient and their healthcare professional, which is not always an option. Hence, a more automated strategy not relying on scheduled visits would be beneficial and necessary to allow a more systematic monitoring.

In general, the technological development continuously provides new and smarter solutions to solve a given task. In recent years, artificial intelligence (AI) has emerged in the medical field due to a desire for increased efficiency in clinical care (Hosny et al., 2018). AI refers to the ability of machines to demonstrate cognitive behavior otherwise associated with humans e.g., solving a given problem (Hessler and Baringhaus, 2018). Different types of AI algorithms exist amongst others deep learning, but they all can find hidden information within a big pool of data. Consequently, these techniques have great potential for assisting healthcare professionals in making evidence-based clinical decisions (Jiang et al., 2017). Moreover, AI algorithms have the potential for determining clinical patterns without involvement of available human expertise and resources.

Medical images are often of complex nature encompassing a lot of information, which can be difficult to extract. Convolutional neural networks (CNNs), which is a subtype of deep learning, have shown great promise for analysis of medical images (Esteva et al., 2017; Anwar et al., 2018; Brinker et al., 2019; Maloca et al., 2019). For this reason, it was speculated whether a CNN-based evaluation of peristomal skin images could provide an alternative approach for objectively monitoring even small changes in progression of PSCs over time. Therefore, the overall aim was to develop a CNN-based algorithm for assessment of peristomal skin images without involvement of human expertise. It was speculated that such AI-based approach would be more objective and consistent in determining the discoloration area compared to current state-of-the-art assessment methods. Secondly, the aim was to develop a similar CNN-based model, which could detect leakage area from images of used ostomy appliances.

In this study, the developed AI models successfully determined the area of peristomal skin discoloration and leakage based on images from peristomal skin and ostomy appliances, respectively. Hence, this is the first time to demonstrate the application of deep learning techniques for evaluation of peristomal skin conditions and leakage patterns and thereby underline the potential for AI algorithms within the ostomy care field.

## Method

Initially, all images were gross sorted on a patient level, and images for each patient were subsequently selected over an evenly distributed timeline. Two models were trained: one based on peristomal skin images (discoloration model) and one based on product images (leakage model). Images were then manually segmented into four distinct classes. For the Discoloration model, the four classes included: Discoloration, Peristomal area, Ostomy, and Background. For the leakage model, the four classes included: Leakage, Ostomy product, Center hole, and Background. A CNN was trained to classify each pixel into one of the four classes. The models were implemented in Python version 3.6.8, Keras version 2.2.4, and Tensorflow version 1.13.1.

### Data

All images were obtained from a specially designed Clinical Trial app on a Nokia Lumia 1520 Windows phone used in two clinical investigations, CP259 (Clinical Trial ID: NCT02517541) and CP300 (Clinical Trial ID: NCT03770078).

For the Discoloration model, 614 peristomal skin images distributed on 56 patients were selected. The images were selected for each patient; thereby, covering the entire time span of the clinical investigation. Each selected image was then manually segmented into four distinct classes: Discoloration, Peristomal area, Ostomy, and Background. The initial segmentation was performed by non-medical personnel after training by medical professionals. Each image segmentation was reviewed by a medical professional and corrected, if needed. The original peristomal skin images had dimensions of 1,280 × 720 × 3 pixels and were scaled to a size of 256 × 256 × 3 pixels to minimize memory load during model training. Images with and without discoloration were used for analysis.

For the Leakage model, 954 product images were selected, and each image had an image segmentation performed by a third-party software system namely JLI Vision Leakage Analyzer^1^ The original product images had dimensions of 2,592 × 1,456 × 3 pixels, which were scaled to a size of 256 × 256 × 3 pixels to minimize memory load during model training. Images with and without leakage were used for analysis.

### Model

Semantic segmentation is a well-known task within deep learning and a multitude of different network architectures have been used to perform this task (Garcia-Garcia et al., 2018). Initially, an empirical network architecture test was performed where multiple architectures were tested. The network with the most promising results was chosen for subsequent use, which turned out to be a U-NET architecture (Ronneberger et al., 2015; Iglovikov and Shvets, 2018).

As the dataset was relatively small and since the task of manual segmenting images are a costly procedure, the concept of transfer learning was applied. This procedure is commonly used in deep learning and weights from a pre-trained network are used for initialization rather than using randomly initiated weights. Commonly, networks trained on the ImageNet (Russakovsky et al., 2014) dataset are used for the initialization. In this way, the learning procedure can be performed on the entire network or it can be chosen to only update the weights on the non-pretrained layers of the network.

The transfer learning was applied to the encoding layers using pre-trained weights from the VGG16 network (Simonyan and Zisserman, 2014) trained on the ImageNet dataset. The weights were imported from the Keras functional API^2^, and the network was trained for 200 epochs with a batch size of 16. The Adam optimizer^3^ was used with a learning rate of 10^−5^, and a categorical cross entropy loss function^4^ was used. The final layer in the model contained a softmax activation function, which outputs a probability for each pixel belonging to a specific class. The sum of probability for a given pixel will always summate to 1. The model architecture and training setting were used for both Discoloration and the Leakage model.

### Validation

The models were evaluated using Precision, Recall, and Accuracy metrics. A Precision Recall Receiver Operating Characteristic curve were obtained by thresholding the classifier output at different values and compute the precision and recall (data not shown). The results were also manually validated by healthcare professionals as a part of the model validation process. In details, the images were screened before and after training by two clinical experts including one nurse. Random samplings of the model predictions were checked by three clinical experts and a panel of skin experts encompassing two dermatologists and three highly experienced stoma care nurses.

A True Positive was defined as a pixel of a class classified as that class (e.g., an Ostomy pixel classified as an Ostomy pixel). A True Negative was defined as a pixel of one of the three other classes not being classified as that specific class (e.g., a Background-, Peristomal area-, or Discoloration pixel not classified as an Ostomy pixel).

The accuracy was calculated by flattening all images into an array of size, where N was the number images predicted and W was the width of images in pixels, H was the height of images in pixels, and C was the total number of classes:

$$\begin{array}{r} \left[\left(N_{image}\;*\;W_{pixels}\;*\;H_{Pixels}\right),\;C\right] \end{array}$$

An index was created using the numpy argmax^5^ function in python. The index of 0 corresponded to the first class, the index of 1 corresponded to the second class etc. From this, an accuracy was calculated for each class using the sklearn.metrics accuracy_score^6^ function.

### Discoloration Intensity

The discoloration intensity was calculated as a post-processing step. Specifically, the discoloration intensities were calculated using all three color channels in an RGB color space using the formula:

$$\begin{array}{r} DiscolorationIntensity=abs\left(R-\left(\frac{G+B}{2}\right)\right) \end{array}$$

Here, R corresponded to the Red channel of the RBG image, G corresponded to the Green channel of the RGB image, and B corresponded to the blue channel of the RGB image.

### Ethical Statement

Images used for development of the deep learning algorithms were derived from clinical investigations performed in accordance with the Declaration of Helsinki of 1964. Images were obtained from two clinical investigations, which were approved by the local ethics committee. Specifically, the clinical investigations were approved by “The Danish National Committee on Health Research Ethics” in Denmark, “The Regional Committees for Medicinal and Health Research Ethics” in Norway, “The Independent Ethics Committee of the Foundation of Evaluation of Ethics in Biomedical Research” in The Netherlands, “The Health Research Authority” in the United Kingdom, and “Salus IRB” in the United States. Written informed consent was obtained for all participating subjects.

## Results

### Discoloration Model

The discoloration model performed image segmentation; subsequently, resulting in each image being divided into four different classes. These classes included the peristomal area, the ostomy itself, the discolored skin, and background. In the model output, the highest probability for a given pixel results in the pixel belonging to that class. A binary mask can then be generated for the class by choosing pixels with highest probability. A yellow marking of a pixel in the binary mask is a 1, meaning the pixel belongs to that class. A purple marking of a pixel in the binary mask is a 0, meaning the pixel does not belong to that class.

**Figure 1:**
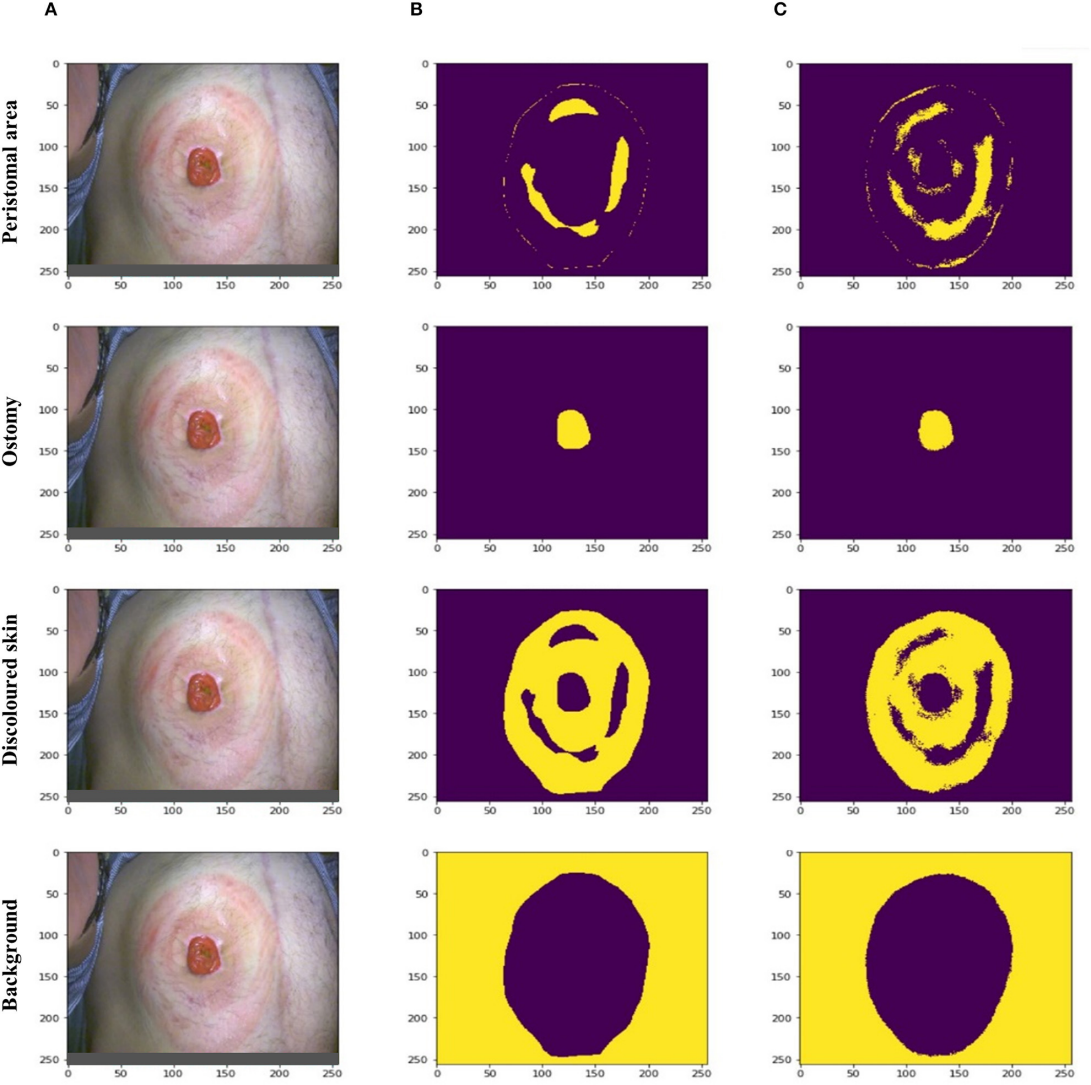
Examples of discoloration model predictions. **(A)** A representative peristomal skin image with varying degree of discolored skin. **(B)** Manual segmentation of the peristomal skin image into four different area classes including the peristomal area, ostomy itself, discoloration area, and background. Yellow marking indicated that the pixel belonged to the given class, while purple indicated that the pixel did not belong to the class. **(C)** Automated segmentation of the peristomal skin image into the same four classes performed by the discoloration model.

A representative example of a peristomal skin image with varying degree of discolored peristomal skin is depicted in Figure 1A. Manual segmentation of the image into the four different classes is shown in Figure 1B, while Figure 1C depicts how the discoloration model divided the image into the peristomal area, ostomy itself, discolored skin, and background. Across the four classes, the areas predicted by the model (Figure 1C) visually appeared like the areas manually marked (Figure 1B); thus, indicating that the model performed well (Figures 1B,C).

### Performance of the Discoloration Model

The performance of the discoloration model was evaluated on three different parameters namely accuracy, precision, and recall (sensitivity) by comparing manually marked and predicted areas. Each of these parameters was calculated for all four classes as depicted in Table 1. The discoloration class alone showed a 95.0% accuracy, and despite being the lowest scoring class, prediction of discoloration area still demonstrated a precision and recall score of 79.6 and 75.0%, respectively (Table 1). Detection of the peristomal area showed an accuracy score of 93.3% with precision and recall scores of 80.2 and 81.1%, respectively. In general, the ostomy itself and the background were the two highest scoring classes across all parameters.

**Table 1:** Summarized performance of the discoloration model.

**Class**	**Accuracy (%)**	**Precision (%)**	**Recall (%)**
Discoloration	95.0	79.6	75.0
Peristomal area	93.3	80.2	81.1
Ostomy	99.7	95.8	96.3
Background	97.1	97.6	98.2

*The model performance was evaluated for each of the four classes: the discoloration area, the peristomal area, the ostomy itself, and the background. Accuracy, precision, and recall scores were calculated for all four classes*.

### Leakage Model

While the discoloration model predicted discoloration on peristomal skin images, the leakage model predicted leakage patterns based on ostomy product images. For the leakage model, each image was divided into four classes including the area of the ostomy product, the center hole, the leakage area, and the background. Again, a yellow color in the visualizations was indicative of the pixel belonging to the specific class, while a purple color indicated that the pixel did not belong to the class in question.

A representative product image was used as an example to demonstrate the outcome of the leakage model (Supplementary Figure 1A). The product image was manually marked, and Supplementary Figure 1B depicts the four classes corresponding to the manual assessment. Next, the leakage model predicted the same four classes with the outcome shown in Supplementary Figure 1C. For all four classes, there was an agreement between the manual annotation and model predictions (Supplementary Figures 1B,C).

### Performance of the Leakage Model

The performance of the leakage model was evaluated by comparing the manually marked and predicted classes. For each of the classes, three different performance parameters were calculated including accuracy, precision, and recall as depicted in Table 2. Prediction of leakage area demonstrated the second highest accuracy score among the four classes (98.8%) alongside the lowest recall and precision scores; 71.5% for both parameters (Table 2). Predictions of the ostomy product area showed an accuracy of 96.1% with precision and recall scores of 89.3 and 91.7%, respectively (Table 2). Overall, predictions of the center hole and the background showed the highest scores across all performance parameters.

**Table 2:** Summarized performance of the leakage model.

**Class**	**Accuracy (%)**	**Precision (%)**	**Recall (%)**
Leakage	98.8	71.5	71.5
Ostomy product	96.1	89.3	91.7
Center hole	99.5	91.5	86.4
Background	96.1	97.2	98.2

*The model performance was evaluated for each of the four classes: the leakage area, the ostomy product, the center hole, and the background. Accuracy, precision, and recall scores were calculated for all four classes*.

### Discoloration Intensity

**Figure 2:**
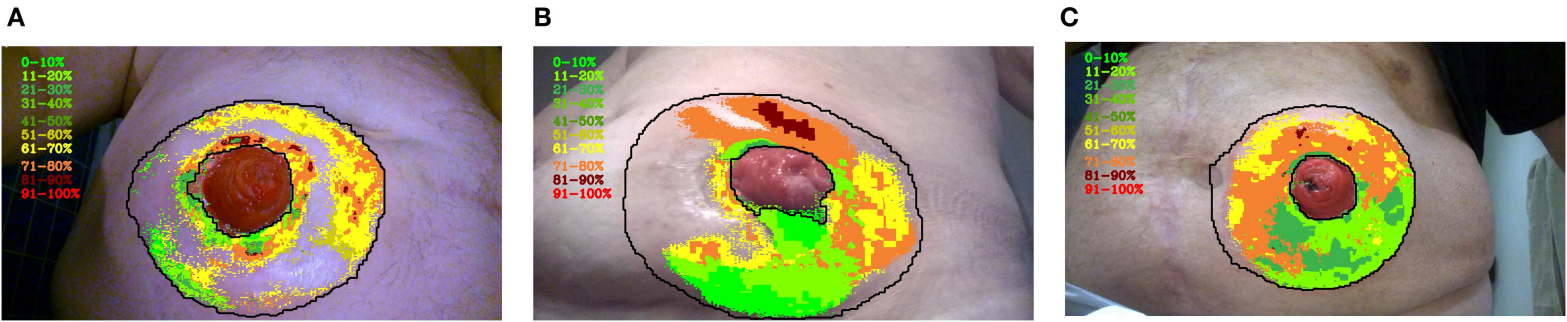
The intensity of peristomal skin discoloration. Three different examples **(A–C)** of peristomal skin images with the overlaid discoloration intensity scores are shown. Each discoloration pixel was divided into brackets with respect to the maximum redness value. The maximum redness intensity was defined as the average redness value of the ostomy itself. The lower redness intensity limit was defined as the minimum value of redness in the discoloration area. The resulting image has discoloration marked in brackets ranging from 0–10, 11–20, 21–30, 31–40, 41–50, 51–60, 61–70, 71–80, 81–90, 91–100%. The color code progresses from green to red as the skin discoloration becomes more intense.

The discoloration intensity was calculated as an overlay to the discoloration model, where 100% intensity of discoloration was defined as the average redness value of the ostomy itself. This resulted in an output image divided into segments of 10% redness intensity ranging from low (green) to high (red). Figure 2 shows three representative peristomal skin images segmented into the 10 different discoloration intensity zones.

In the first example, the discolored skin primarily appeared close to the edges of the peristomal area and close to the ostomy itself (Figure 2A). Some intense, yet sporadic, areas of discolored skin were found close to the ostomy with an intensity score of 81–90%. Despite this, a substantial fraction of the peristomal skin was not classified by the model as being discolored (Figure 2A). In the second example, a larger proportion of the peristomal skin was classified as discolored. While the skin area below the ostomy only had a low intensity of discoloration (0–20%), a large fraction above the ostomy displayed more intense discoloration ranging from 71 to 90% (Figure 2B).

Finally, the last example depicts a peristomal skin image, where the model annotated most of the peristomal area as discoloration to various intensity degrees (Figure 2C). Again, the area above the ostomy appeared to have the most intense discoloration ranging from 61 to 80%. Although a large proportion of the peristomal skin area was annotated as discolored, only a small fraction of the skin reached an intensity score of 81–90% (Figure 2C).

## Discussion

AI in health care is a continuously growing field with several branches starting to implement deep learning algorithms. Among other fields, AI has been applied for image analysis in diabetic retinopathy (Gulshan et al., 2016), stroke and neuroimaging (Rehme et al., 2015; Kamnitsas et al., 2017), skin cancer (Esteva et al., 2017), breast cancer (Sun et al., 2017), interstitial lung disease (Anthimopoulos et al., 2016), Alzheimer's disease (Oh et al., 2019), and eye structures (Maloca et al., 2019). In addition, the number of studies demonstrating a CNN-based approach for wound image segmentation is gradually increasing (Ohura et al., 2019), and AI models have gained popularity for long-term predictions of the wound healing process (Wang et al., 2015; Jung et al., 2016).

AI algorithms can provide accurate, reproducible, and objective methods for medical imaging analysis (Liu and Salinas, 2017; Hosny et al., 2018). In contrast to manual image segmentation, deep learning algorithms can learn directly from the data without the need for involvement of human expertise. Both the discoloration and the leakage algorithms generated in this study were based on supervised learning; a branch of machine learning, where functions and relationships are deducted from labeled training data (Hosny et al., 2018). Such supervised learning is commonly used in healthcare for medical imaging analyses, as it provides more clinically relevant output compared to unsupervised learning (Jiang et al., 2017; Ohura et al., 2019). Although digital analysis of peristomal skin images has been attempted (Iizaka et al., 2014), to the best of our knowledge, no studies has previously demonstrated the usefulness of applying AI algorithms for evaluation of peristomal skin conditions and leakage patterns.

Accuracy is a parameter often used for evaluation of model performance. The two models presented here demonstrated high accuracy scores across all four classes in the combined range of 93.3–99.7%. A study involving a dataset of pressure ulcer images demonstrated a CNN-based model with an accuracy of 0.998 for wound segmentation (Ohura et al., 2019), while a review evaluating the performance of CNN-based methods for medical image analysis in various clinical settings reported accuracy scores ranging from 75 to 99.77% (Anwar et al., 2018). Taken together, the leakage- and discoloration model demonstrated accuracies within the high end of the acceptable range compared to what is reported for AI-based algorithms for medical analysis.

Depending on the model, evaluating performance solely based on accuracy might not be adequate. For the discoloration model, most pixels in the training images did not belong in the discoloration class; hence, the algorithm is slightly biased toward predicting non-discoloration. For this reason, the performance was also evaluated based on precision and recall in conjunction with accuracy. Similarly, the leakage model is biased toward prediction of non-leakage; hence, this model was also evaluated based on precision and recall alongside the accuracy scores. Compared to the other classes in the model, detection of the discoloration area showed lower scores for precision and recall; 79.6 and 75.0%, respectively.

Likewise, prediction of the leakage area showed the lowest recall and precision among the four classes (71.5% for both parameters). These slightly lower performance scores therefore indicated that prediction of discoloration and leakage area were more difficult to correctly annotate than e.g., the ostomy or the center hole. Importantly, AI algorithms often require millions of observations before reaching acceptable performance scores (Obermeyer and Emanuel, 2016), which is a much larger dataset than what was available for developing the algorithms presented here. The precision and recall scores presented here will inevitable need further improvement before these algorithms may replace health care staff. For this reason, inclusion of more peristomal skin- and product images e.g., obtained in clinical trials are needed to enable improvement of the recall and precision scores for the discoloration- and leakage model, respectively.

The Ostomy Skin Tool is the current state-of-the art for assessing a patient's peristomal skin condition. Validation of the tool was based on 20 nurses' assessments of 30 different images of PSCs. When investigating reproducibility and repeatability of the Ostomy Skin Tool, a high intra-nurse agreement (κ = 0·84) was demonstrated, while the inter-nurse agreement was only classified as ‘moderate to good' (κ = 0·54). Specifically, detection of discoloration showed an inter-nurse agreement of κ = 0·59 (Jemec et al., 2011). Based on this validation, it was concluded that the Ostomy Skin Tool was good for monitoring an individual patient by the same nurse, while the outcome from different assessors showed more discrepancies and was therefore considered less reliable (Jemec et al., 2011). In contrast, the two AI-based models presented here showed relatively high precision and recall scores; thus, these models have an advanced level of consistency and reproducibility compared to inter-nurse assessments and provide another level of objectivity.

Importantly, these AI-based models also allow both the patients and the nurses to closely monitor even small changes in PSCs over time; something which would otherwise only be possible, if the patient visited their healthcare professional frequently. Such close and systematic monitoring combined with the models' high level of objectivity and consistency are over time likely to give a clearer picture of the actual prevalence of PSCs. For AI algorithms to achieve more widespread clinical acceptance, further optimization and awareness are needed (Liu and Salinas, 2015). Specifically, more clinical evidence is desirable to fully elucidate the potential and impact of AI models in various medical fields. For the discoloration and leakage area assessment algorithms, we are still in the early phases. Hence, the use of AI in future clinical studies and daily practices among health care professionals will help to unlock and evaluate the true potential for these algorithms within the ostomy care field.

In conclusion, this study provided proof-of-concept that it is possible to utilize AI algorithms for analysis of discoloration and leakage. The implementation of AI algorithms for medical image analysis has great potential for future use. Here, AI-based models showed promising results for objective and consistent assessment of peristomal skin- and ostomy product images aimed at detecting discoloration- and leakage area, respectively. The deep learning techniques presented in this study allow healthcare professionals to closely monitor even small changes in discoloration of the peristomal skin and leakage patterns over time, and these strategies are considered more consistent and objective than the current state-of-the-art skin assessment tool. Although the models performed well on all parameters including accuracy, precision, and recall; AI algorithms continuously improve their performance as more input data is provided and various layers added. Obtaining further data and hands on experience from daily clinical practice will be important to fully elucidate the potential of a deep learning-based automated assessments of peristomal skin discoloration and leakage patterns.

## Data Availability Statement

The raw data supporting the conclusions of this article will not be made publicly available by the authors due to ethical reasons.

## Ethics Statement

The studies involving human participants were reviewed and approved by The Danish National Committee on Health Research Ethics' in Denmark, The Regional Committees for Medicinal and Health Research Ethics in Norway, The Independent Ethics Committee of the Foundation of Evaluation of Ethics in Biomedical Research in The Netherlands, The Health Research Authority in the United Kingdom, and Salus IRB in the United States. The patients/participants provided their written informed consent to participate in this study. Written informed consent was obtained from the individual(s) for the publication of any potentially identifiable images or data included in this article.

## Author Contributions

NA developed the algorithms behind the deep learning models. All authors contributed to the article and approved the submitted version.

## Conflict of Interest

NA is an employee at VENZO.Nxt. PT, NH, and ZS are employees at Coloplast A/S.

## Footnotes

^1^Available online at: https://jlivision.com/

^2^Available online at: https://keras.io/applications/#vgg16

^3^Available online at: https://keras.io/optimizers/#adam

^4^Available online at: https://keras.io/backend/#categorical_crossentropy

^5^Available online at: https://numpy.org/doc/stable/reference/generated/numpy.argmax.html

^6^Available online at: https://github.com/scikit-learn/scikit-learn/blob/fd237278e/sklearn/metrics/_classification.py#L125

## References

[B1] Anthimopoulos M. Christodoulidis S. Ebner L. Christe A. Mougiakakou S. (2016). Lung pattern classification for interstitial lung diseases using a deep convolutional neural network. IEEE Trans. Med. Imaging 35, 1207–1216. 10.1109/TMI.2016.2535865 26955021

[B2] Anwar S. M. Majid M. Qayyum A. Awais M. Alnowami M. Khan M. K. (2018). Medical image analysis using convolutional neural networks: a review. J. Med. Syst 42, 226–226. 10.1007/s10916-018-1088-1 30298337

[B3] Brinker T. J. Hekler A. Enk A. H. Berking C. Haferkamp S. Hauschild A.. (2019). Deep neural networks are superior to dermatologists in melanoma image classification. Eur. J. Cancer 119, 11–17. 10.1016/j.ejca.2019.05.023 31401469

[B4] Burch J. (2011). Peristomal skin care and the use of accessories to promote skin health. Br. J. Nurs. 20:S4. 10.12968/bjon.2011.20.Sup3.S4 21537268

[B5] Esteva A. Kuprel B. Novoa R. A. Ko J. Swetter S. M. Blau H. M.. (2017). Dermatologist-level classification of skin cancer with deep neural networks. Nature 542, 115–118. 10.1038/nature21056 28117445 PMC8382232

[B6] Fellows J. Forest Lalande L. Martins L. Steen A. Størling Z. M. (2017). Differences in ostomy pouch seal leakage occurrences between north American and european residents. J. Wound Ostomy Contin. Nurs. 44, 155–159. 10.1097/WON.0000000000000312 28267122

[B7] Garcia-Garcia A. Orts-Escolano S. Oprea S. Villena-Martinez V. Martinez-Gonzalez P. Garcia-Rodriguez J. (2018). A survey on deep learning techniques for image and video semantic segmentation. Appl. Soft Comput. 70, 41–65. 10.1016/j.asoc.2018.05.018

[B8] Gulshan V. Peng L. Coram M. Stumpe M. C. Wu D. Narayanaswamy A.. (2016). Development and validation of a deep learning algorithm for detection of diabetic retinopathy in retinal fundus photographs. JAMA 316, 2402–2410. 10.1001/jama.2016.17216 27898976

[B9] Herlufsen P. Olsen A. G. Carlsen B. Nybaek H. Karlsmark T. Laursen T. N.. (2006). Study of peristomal skin disorders in patients with permanent stomas. Br. J. Nurs. 15, 854–862. 10.12968/bjon.2006.15.16.21848 17108855

[B10] Hessler G. Baringhaus K. H. (2018). Artificial intelligence in drug design. Molecules 23:2520. 10.3390/molecules23102520 30279331 PMC6222615

[B11] Hosny A. Parmar C. Quackenbush J. Schwartz L. H. Aerts H. J. W. L. (2018). Artificial intelligence in radiology. Nat. Rev. Cancer 18, 500–510. 10.1038/s41568-018-0016-5 29777175 PMC6268174

[B12] Iglovikov V. Shvets A. (2018). TernausNet: U-Net with VGG11 Encoder Pre-Trained on ImageNet for Image Segmentation. Available online at : https://ui.adsabs.harvard.edu/abs/2018arXiv180105746I (accessed January 01, 2018).

[B13] Iizaka S. Asada M. Koyanagi H. Sasaki S. Naito A. Konya C.. (2014). The reliability and validity of color indicators using digital image analysis of peristomal skin photographs: results of a preliminary prospective clinical study. Ostomy Wound Manage 60, 12–29. 24610557

[B14] Jemec G. B. Martins L. Claessens I. Ayello E. A. Hansen A. S. Poulsen L. H.. (2011). Assessing peristomal skin changes in ostomy patients: validation of the ostomy skin tool. Br. J. Dermatol. 164, 330–335. 10.1111/j.1365-2133.2010.10093.x 20973766

[B15] Jiang F. Jiang Y. Zhi H. Dong Y. Li H. Ma S.. (2017). Artificial intelligence in healthcare: past, present and future. Stroke Vasc. Neurol. 2, 230–243. 10.1136/svn-2017-000101 29507784 PMC5829945

[B16] Jung K. Covington S. Sen C. K. Januszyk M. Kirsner R. S. Gurtner G. C.. (2016). Rapid identification of slow healing wounds. Wound Repair Regener. 24, 181–188. 10.1111/wrr.12384 26606167 PMC4820011

[B17] Kamnitsas K. Ledig C. Newcombe V. F. J. Simpson J. P. Kane A. D. Menon D. K.. (2017). Efficient multi-scale 3D CNN with fully connected CRF for accurate brain lesion segmentation. Med. Image Anal. 36, 61–78. 10.1016/j.media.2016.10.004 27865153

[B18] Liu N. T. Salinas J. (2015). Machine learning in burn care and research: a systematic review of the literature. Burns 41, 1636–1641. 10.1016/j.burns.2015.07.001 26233900

[B19] Liu N. T. Salinas J. (2017). Machine learning for predicting outcomes in trauma. Shock 48, 504–510. 10.1097/SHK.0000000000000898 28498299

[B20] Maloca P. M. Lee A. Y. de Carvalho E. R. Okada M. Fasler K. Leung I.. (2019). Validation of automated artificial intelligence segmentation of optical coherence tomography images. PLoS ONE 14:e0220063. 10.1371/journal.pone.0220063 31419240 PMC6697318

[B21] Martins L. Ayello E. A. Claessens I. Steen Hansen A. Hentze Poulsen L. Sibbald R. G.. (2010). The ostomy skin tool: tracking peristomal skin changes. Br. J. Nurs. 19, 960–934. 10.12968/bjon.2010.19.15.77691 20966862

[B22] Meisner S. Lehur P.-A. Moran B. Martins L. Jemec G. B. E. (2012). Peristomal skin complications are common, expensive, and difficult to manage: a population based cost modeling study. PLoS ONE 7:e37813. 10.1371/journal.pone.0037813 22679479 PMC3359986

[B23] Obermeyer Z. Emanuel E. J. (2016). Predicting the future - big data, machine learning, and clinical medicine. N. Engl. J. Med 375, 1216–1219. 10.1056/NEJMp1606181 27682033 PMC5070532

[B24] Oh K. Chung Y.-C. Kim K. W. Kim W.-S. Oh I.-S. (2019). Classification and visualization of Alzheimer's disease using volumetric convolutional neural network and transfer learning. Sci. Rep. 9:18150. 10.1038/s41598-019-54548-6 31796817 PMC6890708

[B25] Ohura N. Mitsuno R. Sakisaka M. Terabe Y. Morishige Y. Uchiyama A.. (2019). Convolutional neural networks for wound detection: the role of artificial intelligence in wound care. J. Wound Care 28, S13–S24. 10.12968/jowc.2019.28.Sup10.S13 31600101

[B26] Rehme A. K. Volz L. J. Feis D. L. Bomilcar-Focke I. Liebig T. Eickhoff S. B.. (2015). Identifying neuroimaging markers of motor disability in acute stroke by machine learning techniques. Cerebral Cortex. 25, 3046–3056. 10.1093/cercor/bhu100 24836690

[B27] Ronneberger O. Ficher P. Brox T. (2015). U-Net: Convolutional Networks for Biomedical Image Segmentation. Available online at: https://arxiv.org/abs/1505.04597 (accessed July 09, 2020).

[B28] Russakovsky O. Deng J. Su H. Krause J. Satheesh S. Ma S. (2014). ImageNet Large Scale Visual Recognition Challenge. Available online at: https://ui.adsabs.harvard.edu/abs/2014arXiv1409.0575R (accessed September 01, 2014).

[B29] Simonyan K. Zisserman A. (2014). Very Deep Convolutional Networks for Large-Scale Image Recognition. Available online at: https://ui.adsabs.harvard.edu/abs/2014arXiv1409.1556S (accessed September 01, 2014).

[B30] Sun W. Tseng T.-L. B. Zhang J. Qian W. (2017). Enhancing deep convolutional neural network scheme for breast cancer diagnosis with unlabeled data. Comput. Med. Imaging Graph. 57, 4–9. 10.1016/j.compmedimag.2016.07.004 27475279

[B31] Wang C. Yan X. Smith M. Kochhar K. Rubin M. Warren S. M.. (2015). “A unified framework for automatic wound segmentation and analysis with deep convolutional neural networks,” in Conference Proceedings : Annual International Conference of the IEEE Engineering in Medicine and Biology Society. IEEE Engineering in Medicine and Biology Society. Annual Conference 2015 (Milan), 2415–2418. 10.1109/EMBC.2015.7318881 26736781

