# Supplementary figures and images for: Automated Assessment of Peristomal Skin Discoloration and Leakage Area Using Artificial Intelligence
Source: Front Artif Intell. 2020 Sep 10;3:72. doi: 10.3389/frai.2020.00072 (PMC7861335; doi:10.3389/frai.2020.00072)

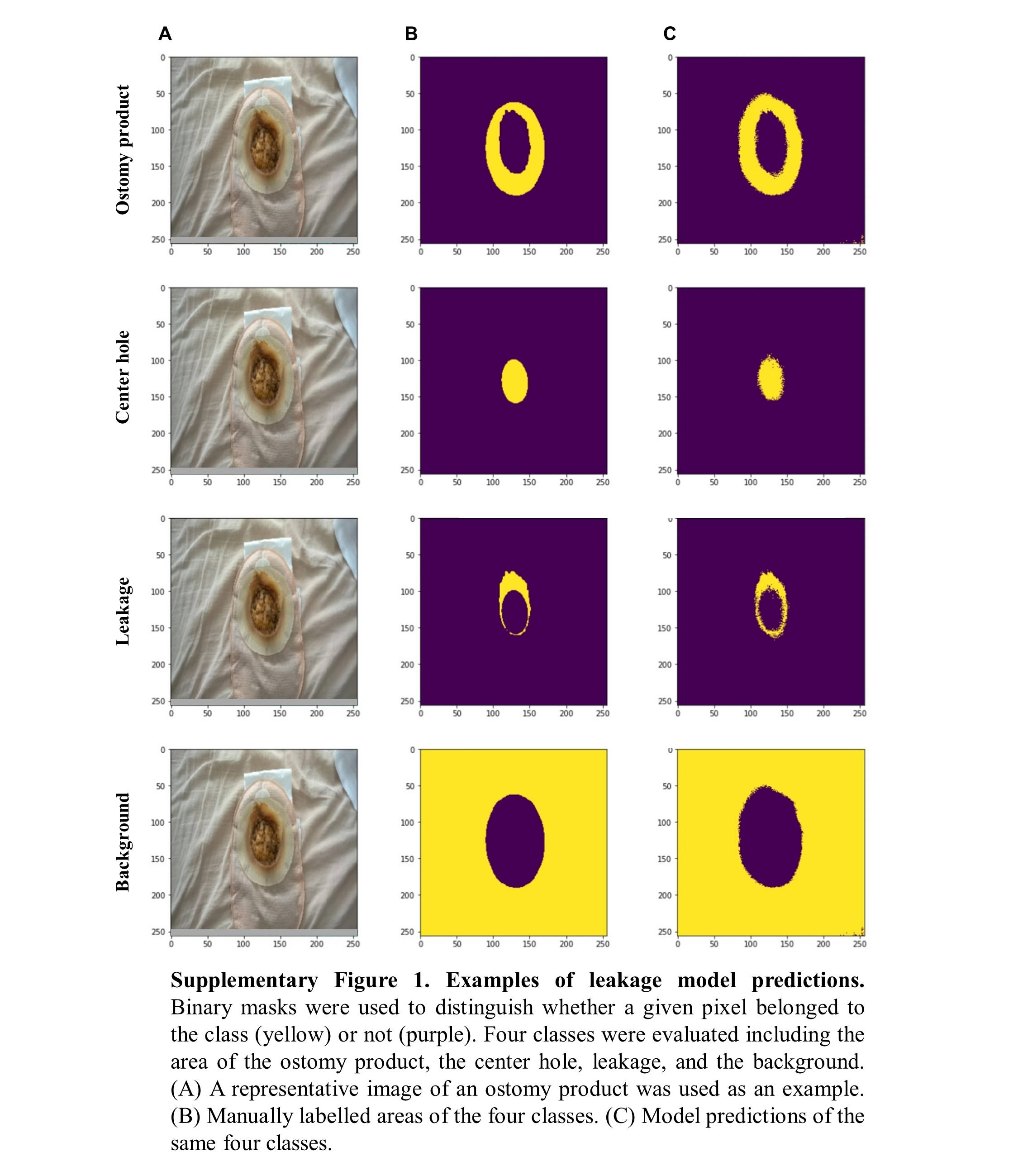

Supplement: Supplementary file 1 [file Image_1.tif]
